# Correction: The impact of reusable tableware packaging combined with environmental propaganda on consumer behaviour in online retail

**DOI:** 10.1371/journal.pone.0295222

**Published:** 2023-11-30

**Authors:** Chao Gu, Jiangjie Chen, Wei Wei, Jie Sun, Chun Yang, Liao Jiang, Jingyue Hu, Baiwan Lv, Shuyuan Lin, Qianling Jiang

The Data Availability link for this paper is incorrect. The correct link is: https://www.researchgate.net/publication/361344644_data_for_the_reusable_tableware_packaging

## References

[1] Gu C, Chen J, Wei W, Sun J, Yang C, Jiang L, et al. (2022) The impact of reusable tableware packaging combined with environmental propaganda on consumer behaviour in online retail. PloS ONE 17(3): e0264562. 10.1371/journal.pone.0264562 35275917 PMC8916672

