# Supplementary material for: The impact of reusable tableware packaging combined with environmental propaganda on consumer behaviour in online retail
Source: PLoS One. 2022 Mar 11;17(3):e0264562. doi: 10.1371/journal.pone.0264562 (PMC8916672; doi:10.1371/journal.pone.0264562)
Supplement: S1 File — (PDF) [file pone.0264562.s001.pdf]

ELSEVIER

Certificate of Elsevier Language Editing Services

The following article was edited by Elsevier Language Editing Services:

"The impact of reusable tableware packaging combined with environmental propaganda on consumer behavior in online retail"

Authored by:

Chao Gu

Date: 06-Dec-2021
Serial number: LE-227295-E1B041AD8850
