# Supplementary material for: The impact of reusable tableware packaging combined with environmental propaganda on consumer behaviour in online retail
Source: PLoS One. 2022 Mar 11;17(3):e0264562. doi: 10.1371/journal.pone.0264562 (PMC8916672; doi:10.1371/journal.pone.0264562)
Supplement: Attachment — Submitted filename: author to respond reviewer2.docx [file pone.0264562.s002.docx]

Response to Reviewer 2 Comments

Dear Referee,

Thank you very much for the valuable suggestions, which have made our paper more comprehensive. We have responded to each of your comments below:

**Point 1:** - The title of the manuscript is too lengthy - consider making it concise. Perhaps may remove "Take ceramic chopsticks for example".

**Response 1:** Thank you for the suggestion. We changed the title of the manuscript. The new title is more specific and concise.

**Point 2:** - The manuscript is poorly written with rampant grammatical errors. Some of the sentences are not clear and do not flow well. I suggest the authors to send for professional proofreading and copy editing services.

**Response 2:** Thank you for the suggestion. We used the Professional English Language Editing service for this revision. The certificate would be submitted in supplementary materials.

**Point 3:** - Avoid using is "isn't", "can't" etc. as this is a formal academic writing.

**Response 3:** Thank you for the suggestion. We focused on these informally written words and adjusted the presentation.

**Point 4:** - Some in-text citations are not done according to the right format (i.e. surname of authors).

**Response 4:** Thank you for the suggestion. We re-edited the references to the article with endnote.

**Point 5:** - Citations need to be updated with recent literature of within 5 years - i.e. Hamilton (1974); Stevels, Agema, & Hoedemaker, 2001; Gardner & Levy (1955) are too old.

**Response 5:** Thank you for the suggestion. We have deleted the earlier references. At the same time, the newly added literatures are all recent.

**Point 6:** - Why section 3.2.2 Brand Image (BI) is suddenly being discussed together in Method section. Where are the rest of the Literature Review of the other focal constructs?

**Response 6:** Thank you for the suggestion. We adjusted the literature review of each construct to the second section and discussed them one by one. The content of literature review is supplemented. The revised part has been marked in red.

**Point 7:** - "The marketing benefits of incorporating environmental propaganda into the Reusable Tableware packaging can be influenced by the brand." -- sentence not clear at all of what the author actually meant here.

**Response 7:** Thank you for the suggestion. It means that different brands may interfere with our evaluation of the marketing effectiveness of Reusable Tableware packaging. We have readjusted the statement to make it easier to understand. The revised part has been marked in red.

**Point 8:** - Need to add a section on Packaging literature review.

**Response 8:** Thank you for the suggestion. We have added a separate sub-section on Packaging Literature Review in section 2. The revised part has been marked in red.

**Point 9:** - Sampling and clustering method of ceramic chopsticks are quite confusing.

**Response 9:** Thank you for the suggestion. We rearranged the order of discussion in the Method section, deleted and added some content to make the research method more clear. In addition, we have added a figure in Section 3 to illustrate the research procedure (Figure 1). The revised part has been marked in red.

**Point 10:** - Why focus group meetings is included rather than experimental design alone should be further justified, and results of the focus group meetings should be reported.

**Response 10:** Thank you for the suggestion. The reason for conducting focus group interview is that when selecting representative samples of each group, we need to consider the common characteristics of the group and the design advantages of each picture, which requires professional advice. Therefore, we invited four experts to form a focus meeting for discussion. In Section 4, we supplemented the reasons for the experts' discussion and selection process of the final representative sample (Table 6). The revised part has been marked in red.

**Point 11:** - Should explain the development of propaganda poster in promoting environmental protection - is this poster for experiment being guided by specific marketing model/theory on advertising? What is the process involved when you come up with the propaganda poster?

**Response 11:** Thank you for the suggestion. Those two propaganda posters used for chopstick brand promotion are not designed by us. They were selected from existing posters through a series of classification and surveys. We adjusted the description of the selection process to make it clearer. This process includes eight steps, as follows:

1. In the initial stage of the study, 200 posters of environmental protection were collected from the Internet

2. 30 of them were selected by random numbering, and the respondents were invited to classify

3. Multivariate scale analysis was carried out on the classification results, and the 6-dimensional coordinates of each poster were calculated

4. MANOVA was used to compare the coordinates of each group to confirm the number of the six groups was optimal

5. The accuracy of clustering results was confirmed by comparing various cluster analysis methods

6. An expert focus group interview was held to select representative samples from each group

7. The environmental propaganda satisfaction of the 6 posters was investigated by questionnaire

8. There are significant differences between the two samples with the highest and lowest satisfaction which were examined by ANOVA

**Point 12:** - Measurement Item for Brand Image - 2.This brand of ceramic chopsticks has a clean image -- not clear what you meant by "clean image"? This is a really confusing phrase from the marketing perspective when you are trying to describe a brand image.

**Response 12:** Thank you for the suggestion. As for the literature on brand image, we quoted the existing scale in previous studies without changing the expression. The items are derived from Sasmita, J.; Suki, N.M. Young Consumers' Insights on Brand Equity: Effects of brand association, brand loyalty, brand awareness, and brand image. International Journal of Retail & Distribution Management 2015. In addition, because our respondents are Chinese, the clean brand image expresses the image of purity and sincerity in the Chinese context. In section 3, we supplement explanations of the original literature to illustrate the meanings of these items. The revised part has been marked in red.

**Point 13:** - Measurement Item for Brand Image - 3. This brand of ceramic chopsticks has a differentiated image in comparison with the other brand -- not clear what is "differentiated image"? You are using an important construct from marketing, yet you have wrongly defined them. Please use proper literature on branding to help you in operationalizing brand image.

**Response 13:** Thank you for the suggestion. The brand image items in the article were all quoted from the same literature, and we did not change the expression. We reexamined the original literature and differentiated image here means that consumers believe that a brand's brand image is different or special from other brands. We admit that the item we cited is just one of many literatures that attempt to define brand image. Brand image is a frequently discussed concept, and there are various descriptions about it. In Section 3, we supplement explanations of the original literature to illustrate the meanings of these items. The revised part has been marked in red.

**Point 14:** - Sampling and experiment design is being discussed in several separated sections of the manuscript (i.e. 3.1 Sample collection and clustering, 3.3 Preparation of experimental samples, 3.2.3 Experimental design, 3.4.4 Experimental design). Please consider re-arranging the experiment design description and making the paragraphs more concise.

**Response 14:** Thank you for the suggestion. In order to make the paragraphs more concise and explain the research more clearly, we have revised the organization of Section 3, including the adjustment of the sequence of paragraphs, addition and deletion of content. The revised part has been marked in red.

**Point 15:** - The overall discussion section is quite shallow and only offering a surface justification. Consider writing in a more critical manner by considering the logical connections of the constructs.

**Response 15:** Thank you for the suggestion. We have added two new paragraphs in Section 5 to try to discuss and explain in more depth. In addition, some statements are adjusted. The revised part has been marked in red.

**Point 16:** - The writing of these particular statements is very confusing with no continuity among one sentence and the next. "The accumulation of time is a necessary factor for re-establishing Brand Loyalty (Schultz & Bailey, 2000). The

strangeness of the new packaging will persist for some time, but it is inevitable when redesigning the product packaging. It should be noted that the package with low EPS significantly reduced Brand Loyalty compared with the original

package." -- not sure what you meant to express here?

**Response 16:** Thank you for the suggestion. What we want to express is that consumers need time to adapt to the new packaging, it does take a process for consumers to become familiar with the new packaging from unfamiliar and re-establish brand loyalty. We have revised this paragraph in section 5. The revised part has been marked in red.
